# The Impact of Family Socioeconomic Status on Elderly Health in China: Based on the Frailty Index

**DOI:** 10.3390/ijerph19020968

**Published:** 2022-01-15

**Authors:** Wenjian Zhou, Jianming Hou, Meng Sun, Chang Wang

**Affiliations:** 1 Northeast Asian Studies College, Jilin University, Changchun 130012, China; zhouwj20@mails.jlu.edu.cn; 2 Center for Northeast Asian Studies, Jilin University, Changchun 130012, China; sunmeng_meng@jlu.edu.cn; 3 School of Physical Education, Northeast Normal University, Changchun 130024, China; wangc358@nenu.edu.cn

**Keywords:** family socioeconomic status, elderly health, frailty index, mediation effect

## Abstract

China is about to enter a moderate aging society. In the process of social and economic development, the family socioeconomic status and health status of the elderly have also changed significantly. Learning the impact of family socioeconomic status on elderly health can help them improve family socioeconomic status and better achieve healthy and active aging. Using the data of the Chinese Longitudinal Healthy Longevity Survey in 2018, this study firstly analyzed the impact of family socioeconomic status on elderly health using the multivariate linear regression model and quantile regression model, the heterogeneity of different elderly groups using subsample regression, and the mediation effects of three conditions associated with the family socioeconomic status of the elderly. The results show that family socioeconomic status has a negative effect on the frailty index, that is, it has a positive impact on elderly health. Family socioeconomic status has a higher positive impact on the health status of the middle and lower age elderly and rural elderly. Overall living status and leisure and recreation status both have mediation effects, while health-care status has no mediation effect.

## 1. Introduction

According to the seventh national census of China, the number of people aged 60 and above in 2020 was 260 million accounting for 18.7% of the total population, and the number of people aged 65 and above was 190 million accounting for 13.5% of the total population [1]. Although China is still in the mild aging stage, it is about to enter a moderate aging society. The scale and proportion of the elderly population will continue to expand. The increasingly serious problem of the aging population not only brings great pressure to the country but also poses various threats to the health and security of the elderly.

With the increase of age, the physiological health of the elderly population is declining, and the mental health issue has become more prominent in recent years. The physical and mental health problems of the elderly bring a series of challenges to the construction of China’s public health system, the construction of an age-friendly society, and the formulation of health standards for the elderly. In particular, China still has a typical dual economic structure at present. The health levels of the elderly population in urban and rural areas differ greatly, and they also face different health risks. At the same time, the socioeconomic status of the elderly begins to decline after a significant shift in their social roles, which often affects their overall living status, leisure and recreation status, health-care status, etc. Quality of life has an intuitive impact on the health status of the elderly. Leisure and recreational activities not only relieve the stress and tension in life, but also help to enhance social interaction among the elderly, maintain the stability of human body functions, and improve the psychological state. Health-care services, as a means of protection when health is at risk, play a last resort role in the health of the elderly.

The Outline of Healthy China 2030 Plan proposed to promote healthy aging and ensure the health of the elderly [2]. The fifth plenary session of the 19th CPC Central Committee approved the CPC Central Committee’s Proposals on Formulating the 14th Five-Year Plan for National Economic and Social Development and the Long-Term Goals of 2035, which proposed to comprehensively promote the construction of a healthy China, give priority to the development of the people’s health, implement the national strategy of actively responding to population aging, and actively develop human resources for the elderly [3]. The implementation of active aging and the development of human resources for the elderly can delay the decline of their socioeconomic status and promote active aging and healthy aging.

In a marriage-based family, the husband and wife are closely interdependent in life and economy. They support each other and share their weal and woe. Both parties determine the socioeconomic status of the entire family, which in turn has a very important impact on both parties. However, existing studies (reviewed in Section 2) have mostly analyzed the effects of older adults’ socioeconomic status on their self-rated health, physical health, and mental health but less on their overall health status from the perspective of family socioeconomic status. Based on the above research backgrounds, it is of great theoretical and practical significance to analyze the impact, heterogeneity, and different mechanisms of the family socioeconomic status of the elderly on their comprehensive health status. Theoretically, it makes up for the shortage of research between family socioeconomic status and elderly health from a comprehensive perspective. Realistically, it calls for more attention to help the elderly improve their family socioeconomic status and comprehensive health status from different dimensions and reduce the inequalities within them, and provides a reference basis for the formulation of public health policy for the elderly, so that they can enjoy happy and healthy lives and successfully achieve healthy and active aging.

## 2. Literature Review

The health production theory suggests that people’s optimal decisions about their health needs are influenced by a variety of factors, including health insurance, lifestyle, education, income status, and living environment [4]. The health causation theory holds that health is also influenced by social structural factors, and socioeconomic status of an individual affects their health status. The higher the socioeconomic status, the better the health status [5]. The social stratification theory assumes that the differences between social groups are universal. People have both natural and social differences. Natural differences are formed by the physical differences of people, whereas social differences are formed by people due to social factors, such as political, economic, cultural, and interaction relationships. It is based on certain criteria for distinguishing people’s positions in social activities and social relations, and the common stratification criteria are economic income, occupation, education level, power, etc. [6]. The existence of social stratification structure leads to the inequality of socioeconomic status and family socioeconomic status.

Socioeconomic status based on the social stratification theory is the social class or position in which an individual or group is located and is a comprehensive reflection of education level, occupational rank, income level, etc. Inequality in socioeconomic status can lead to inequality in one’s own health. Several scholars have revealed this phenomenon through their studies. Winkleby found that socioeconomic status plays a determinant role in almost all diseases and all stages of life [7]. He found that the household income of older adults has a positive effect on their life satisfaction and physical health status and occupational status only has a positive effect on their physical health status, but education has no effect on their life satisfaction and physical health status [8]. Zhang et al. concluded that elderly people with low socioeconomic status, poor income level, low education level, and manual labor-oriented jobs or no job mostly have severely underutilized health services or no health-care coverage at all, leading to the worsening of their own health status [9]. Kuo et al. examined the impact of socioeconomic status on colorectal cancer risk, staging, and survival under the National Health Insurance system in Taiwan [10]. However, these studies mainly analyze the different dimensions of socioeconomic status and lack a comprehensive consideration of socioeconomic status. Some scholars use the socioeconomic status index to study its impact and mechanism on the health of the elderly. Cristine et al. suggested that people with lower socioeconomic status are more likely to be in an unfavorable environment and have negative emotions and potential stress, which in turn have a negative impact on their health [11]. Xue et al. suggested that socioeconomic status affects the physical and mental health of older adults through mediating variables, such as sleep quality, dietary patterns, physical activities, and social participation [12]. Liu et al. found that socioeconomic status positively influences the health of older adults through food access, physical activities, recreational activities, and improving their overall well-being [13]. Wang et al. extended the study of the influence mechanism from a social capital perspective and found that high socioeconomic status groups increase their health advantage through a high frequency of social interactions with friends; low socioeconomic status groups mitigate the health disadvantage caused by low status through social trust [14]. However, these studies do not consider the impact of socioeconomic status from a family perspective.

Family socioeconomic status is a comprehensive reflection of the individual socioeconomic status of family members, and it is the social class or status of family members based on the family cooperation model. Unequal family socioeconomic status can negatively affect education, occupational status, and the health of children. Javier found that the lower the family socioeconomic status of elementary school students, the lower their scores in basic competencies [15]. Meng et al. investigated the family socioeconomic status in China and found that the socioeconomic status of students’ families has important effects and constraints on the students’ preferences with regard to the different types of higher education schools and majors [6]. Zhu et al. argued that the influence of family status on the youth’s attainment of initial position is changing in a wave-like fashion [16]. Cheng et al. found that the family socioeconomic status of secondary school students is significantly and positively related to overall psychological quality and its dimensions [17]. Unequal family socioeconomic status can also lead to inequality in individual’s health. However, only a few studies have focused on this aspect. Huang et al. concluded that a higher family socioeconomic status has a significant contribution to their own health in China [18]. Cao et al. thought consistently higher early family socioeconomic status and upward socioeconomic status mobility will lead to a higher incidence of good health in old age, while continuous lower early family socioeconomic status is the opposite, that is, the impact of early family environment on the elderly health is cumulative [19]. Ghasemi et al. found that subjective perception of family socioeconomic status can explain differences in health-related quality of life of low-income people in Iran [20]. Booysen et al. also found that family structure and family socioeconomic status both have an influence on public health [21]. However, these studies do not clarify how family socioeconomic status affects the comprehensive health status of the elderly.

**Figure 1:**
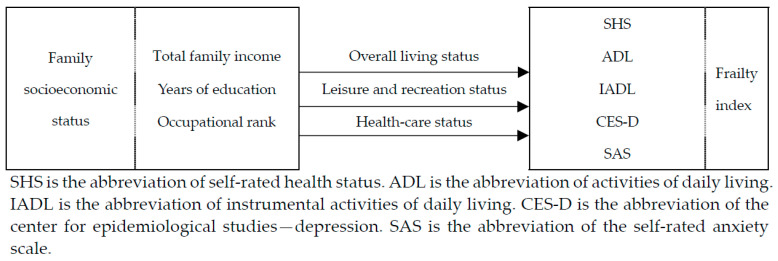
Framework diagram of theoretical analysis.

Based on the above literature analyses, we think that many studies have been conducted on both socioeconomic status and family socioeconomic status, but the existing studies mainly focus on the influence of individual socioeconomic status on the health status in different dimensions of themselves, more on the intergenerational influence of family socioeconomic status on children and less on the impact and mechanism of family socioeconomic status on comprehensive health status. We know that when the socioeconomic status of a family is higher in real life, its members usually enjoy better living conditions, abundant leisure and entertainment activities, and high-quality medical and health services, which will have a positive effect on their health. Therefore, based on the above theories, this study creatively analyzes the impact and mechanism of family socioeconomic status on frailty index. In the present study, the total family income, the comprehensive years of education, and the comprehensive occupational rank before retirement of elderly couples were synthesized into the family socioeconomic status index. The frailty index was used as a measure of the comprehensive health status of the elderly. The impact of family socioeconomic status on elderly health was first analyzed, followed by the differences in the impact of family socioeconomic status on the health status of different elderly groups as well as the possible mechanisms of the impact. Figure 1 shows the specific theoretical analysis framework.

## 3. Study Design

### 3.1. Data

The data used in this study were obtained from the Chinese Longitudinal Healthy Longevity Survey in 2018 (CLHLS was downloaded from https://opendata.pku.edu.cn/dataverse/pku (accessed on 1 November 2021)), which is a national-wide and longitudinal survey of the elderly organized by the Center for Healthy Aging and Development Research/National Development Research Institute of Peking University. The detailed information about the survey design has been reported in previous research [22,23,24]. The samples were collected from 23 provinces of China, and the total number of valid samples was 15,874. The contents of the surviving respondents’ questionnaires included the basic conditions, socioeconomic conditions, and various health conditions of the elderly, which cover all aspects of the elderly and meet the needs of the study. In the present study, elderly people aged 60 and above were included, and 7599 samples were obtained after deleting those samples with missing or invalid variable values.

### 3.2. Variable Descriptions

#### 3.2.1. Explained Variable

The explained variable is the comprehensive health status of the elderly, and the frailty index is used as a measurement method. The frailty index, or cumulative health deficit index, refers to the proportion of health deficit indicators among all measures of health for an individual and can be understood as an accumulation of health deficits. Health deficits can be measured somatically, functionally, and psychologically [25]. The number of variables used to construct the index is not standardized, usually ranging from 30 to 70 and taking values between 0 and 1 [26]. Drawing on previous research results and combining data availability and research objectives, this study selected 32 indicators measuring health status to construct the frailty index, covering self-rated health status (SHS), activities of daily living (ADL), instrumental activities of daily living (IADL), the center for epidemiological studies–depression (CES-D), and the self-rated anxiety scale (SAS). The SHS was assigned in the following manner: 0, “very good”; 0.25, “good”; 0.5, “general”; 0.75, “bad”; and 1, “very bad”. The ADL are reflected by the elderly’s problems in bathing, dressing, toileting, indoor transferring, control of urination and defecation, and feeding (six aspects). For each aspect, if the elderly does not need assistance, a value of 0 was assigned; if the elderly needs one part assistance, a value of 0.5 was assigned; and if the elderly needs more than one part assistance, a value of 1 was assigned. The IADL are reflected by the questions of whether the elderly can go outside to visit neighbors, go shopping, make food, wash clothes, walk 1 km continuously, carry a 5 kg weight, crouch and stand three times continuously, and take public transportation by themselves (eight aspects). For each aspect, if the elderly can do so, a value of 0 was assigned; if the elderly encounters little difficulty, a value of 0.5 was assigned; if the elderly is unable to do so, a value of 1 was assigned. The CES-D consists of 10 questions, with regard to whether the older person is bothered by some small things, has difficulty in concentrating, feels sad or depressed, struggles to do things, has hope for the future, feels nervous or afraid, is as happy as when he or she was young, feels lonely, and feels unable to continue life as well as his or her sleep quality. For the seven questions reflecting negative emotions, the assigned values were as follows: 0, “never”; 0.25, “seldom”; 0.5, “sometimes”; 0.75, “often”; and 1, “always”. For the three questions reflecting positive emotions with regard to whether the elderly has hope for the future and is as happy as when he or she was young as well as his or her sleep quality, they were assigned with the opposite values. The SAS is composed of seven questions with regard to whether the elderly feels uneasy, worried and annoyed, cannot stop or control worry, is worried too much about all kinds of things, is very nervous and finds it difficult to relax, is so anxious that he or she cannot sit still, easily gets annoyed or irritated, and feels as if something terrible is going to happen. We assigned the values according to the frequency of each problem: 0, “never”; 0.33, “for several days”; 0.67, “more than half of days”; and 1, “almost every day”. Finally, the scores of the 32 indicators were summed and then divided by the theoretical maximum score of 32 to obtain the frailty index of each elderly person.

#### 3.2.2. Explanatory Variable

The explanatory variable is the family socioeconomic status of the elderly, which consists of three dimensions: total family income, comprehensive years of education, and comprehensive occupational rank before retirement of the elderly couple (for the currently spouseless elderly, this study used their own years of education and occupational rank before retirement). The total family income is the total income of the whole family in the last year, which was processed logarithmically in this study. The range of years of education is 0–22 years, and those samples with 22 years or more of education were treated as 22 years. For the occupational level before retirement, in accordance with the study of Xue and Ge, the present study defined “professional, technical, governmental, institutional, managerial, and military personnel” as senior practitioners assigned with a value of 3; “commercial, service, and industrial workers” as intermediate practitioners with a value of 2; and the other options as general practitioners with a value of 1 [12]. For the years of education and occupational rank, previous studies have only considered the elderly individuals. Therefore, in the present study, the spouses of the elderly were also taken into consideration. We calculated the comprehensive years of education and occupational rank before retirement of the elderly couples in accordance with Zhu and Li’s study, which enables to evaluate the advantages and disadvantages of the different types of family status more accurately using an approach based on the Pythagorean theorem [16,27]. Finally, the present study used the entropy weight method to synthesize the total family income, the comprehensive years of education, and the comprehensive occupational rank before retirement of elderly couples into a family socioeconomic status index. The entropy weight method is currently the main method of objective assignment method, which aims to assign weights to each evaluation index based on the degree of difference between its values and construct a composite index.

#### 3.2.3. Control Variables

Based on the survey data and existing studies, this study selected the basic personal information and social security status of the elderly as control variables. The control variables included gender, age, marital status, residential area, co-residence mode, number of surviving children, whether or not he or she has retirement pension/public old-age insurance/private or commercial old-age insurance, and whether or not he or she has medical insurance [8,9,12,13,14]. In this study, we classified marital status into without spouse and with spouse, and residential area into urban and rural areas. The co-residence mode included living alone, living with family members, and living in an institution, which are generated into two dummy variables: whether or not living with family members and whether or not living in an institution.

#### 3.2.4. Mediating Variables

The mediating variables were the overall living status, leisure and recreation status, and health-care status of the elderly. In the current study, two dichotomous variables, “is all of the financial support sufficient to pay for daily expenses” and “self-reported quality of life (“very bad”, “bad”, and “general” were merged into “bad”, and “good” and “very good” were merged into “good”),” were selected to construct the overall living status. If the financial support is sufficient and the quality of life is good, the overall living status is “good”; otherwise, it is “bad”. The two dichotomous variables, “whether he or she exercises or not now” and “whether he or she has traveled in the past 2 years”, were selected to construct the leisure and recreation status. If they exercise regularly and have traveled, the leisure and recreation status is good; otherwise, it is “bad”. The two dichotomous variables, “whether he or she can get adequate medical service at present” and “whether he or she has regular physical examination once a year”, were selected to construct the health-care status. If they can get adequate medical service at present and have regular physical examination once a year, the health-care status is good; otherwise, it is “bad”. Table 1 shows the variables and data statistics.

### 3.3. Models

#### 3.3.1. Multiple Linear Regression Model

In the current study, we used the frailty index of the elderly as the explained variable and the family socioeconomic status index as the explanatory variable and added various control variables to establish a multiple linear regression model to analyze the influence of family socioeconomic status on the health of the elderly.

(1) $$Y_{i}=\alpha +\beta_{0}X_{i}+\sum \beta_{j}Z_{ij}$$

In Equation (1), $Y_{i}$ is the frailty index of the *i*th elderly person, and α is a constant term. $X_{i}$ is the explanatory variable, which indicates the family socioeconomic status index of the *i*th elderly person, and $\beta_{0}$ is its coefficient. $Z_{ij}$ is the *j*th control variable of the *i*th elderly person, and $\beta_{j}$ is the coefficient of each control variable.

#### 3.3.2. Quantile Regression Model

Because of the high heterogeneity of the health status of older adults, the same family socioeconomic status may have different effects on older adults with different frailty status. Therefore, we also used the quantile regression model to analyze the effects of family socioeconomic status on frailty indices at different quartiles to verify whether the findings of the multiple linear regression model are still supported.

(2) $$Q_{i\theta }\left(Y_{i}\right)=\alpha +\beta_{0\theta }X_{i}+\sum \beta_{j\theta }Z_{ij}$$

In Equation (2), $Q_{i\theta }\left(Y_{i}\right)$ denotes the conditional quantile of the frailty index for a given distribution of explanatory and control variables, where *θ* denotes the quantiles, and 10%, 25%, 50%, 75%, and 90% are selected in turn. The remaining variables and parameters are explained as in the multiple linear regression model above.

#### 3.3.3. Mediating Effect Model

The mediating variables are the overall living status, leisure and recreation status, and health-care status of the elderly, all of which are dichotomous variables. When the mediating variable is a categorical variable, the mediation effect analysis needs to be conducted by calculating a confidence interval through a two-step regression method. The procedure for testing the mediating effect is as follows:

(3) $$M=aX+\varepsilon_{2}$$

(4) $$Y=c^{\prime }X+bM+\varepsilon_{3}$$

where *Y* denotes the explained variable, *X* denotes the explanatory variable, and *M* denotes the mediating variable. Equation (3) represents the regression of the mediating variable on the explanatory variable, and logistic regression is used. Equation (4) represents the regression of the explained variable on both the explanatory and mediating variables, and linear regression is used. In the present study, we first used the Stata 15.0 software to obtain the estimated values of regression coefficients and robust standard errors of a and b. Then, we used the Medci command in the package of RMediation (downloaded from https://cloud.r-project.org/bin/windows/contrib/3.5/RMediation_1.1.4.zip (accessed on 5 November 2021)) of R 3.5.1 software (R Foundation for Statistical Computing, Vienna, Austria) to conduct the coefficient product distribution test to obtain the confidence interval of the mediating effect [28]. Moreover, if this confidence interval does not contain 0, it indicates the existence of the mediating effect [29].

## 4. Results

### 4.1. The Effect of Family Socioeconomic Status on Elderly Health

A multiple linear regression model was established as a benchmark model to analyze the effect of family socioeconomic status on elderly health. From Model 1 in Table 2, it can be seen that the family socioeconomic status of the elderly has a significantly negative effect on the frailty index at the 1% level, and for every 1 unit increase in the family socioeconomic status, the frailty index decreases by 0.050 units. We performed a multicollinearity test which indicated that the problem of multicollinearity was excluded in the multiple linear regression model.

Because of the high heterogeneity of the health status of older adults, the same family socioeconomic status may have different effects on older adults with different frailty status. Then we also developed quantile regression models to analyze the effects of family socioeconomic status on the elderly health in different quantiles. Models 2–6 in Table 2 show that the effects of family socioeconomic status of older adults on the frailty index remain significantly negative at the 1% level, and the coefficients of the effects are, respectively, −0.041, −0.058, −0.058, −0.055, −0.067.

The results of the multivariate linear regression model and quantile regression models suggest that improving family socioeconomic status can reduce the frailty index and promote the health of the elderly.

### 4.2. Robustness Test

In the current study, the economic status compared with local people, the average years of education, and the average occupational level before retirement of elderly couples are integrated into the replaced family socioeconomic status index using the entropy weight method to conduct a robustness test. Models 7–12 in Table 3 show that the effects of replaced family socioeconomic status of older adults on the frailty index remain significantly negative at the 1% level, and the coefficients of the effects are, respectively, −0.070, −0.050, −0.071, −0.075, −0.081, −0.100. These results also demonstrate that the increase of family socioeconomic status can decrease the frailty index and promote the elderly health, indicating that the empirical results obtained above are reliable.

### 4.3. Heterogeneity Analysis

The above analysis found that both residential area and age have significant influence on the health of the elderly. Therefore, we used the multiple linear regression model to continue to analyze the different effects of family socioeconomic status on the health of the elderly in different residential areas and at different ages.

Table 4 shows that the impacts of the family socioeconomic status of the urban and rural elderly on the frailty index are −0.043 and −0.088, both significant at the 1% level. However, the impact in urban areas is lower than rural areas.

Table 5 shows that the effects of family socioeconomic status on the frailty index for the elderly aged 60–69 and 70–79 years (lower and middle age) are −0.043 and −0.088, both significant at the 1% level, whereas the effect of family socioeconomic status on the frailty index is no longer significant for the elderly aged 80 years and above (higher age).

### 4.4. Mediating Effect Analysis

The level of family socioeconomic status generally directly affects the overall living status, leisure and recreation status, and health-care status of the elderly. Therefore, those were selected as mediating variables to analyze their mediation effects in the influence of family socioeconomic status on elderly health. Table 6 shows the results after adding each mediating variable to the baseline linear regression model. From the comparison with Model 1, it can be seen that the absolute values of the impact coefficients of family socioeconomic status in Models 18–20 are becoming smaller and still significant at the 1% level, and that the impact coefficients of each mediating variable are significantly negative at the 1% level, which initially indicates the existence of mediation effects for each of the above mediating variables.

We further tested the mediation effect by calculating the confidence interval through a two-step regression method. The results in Table 7 show that the 95% confidence intervals of the estimated mediation effects for the overall living status and leisure and recreation status are, respectively, [−0.116, −0.074] and [−0.184, −0.127]; they do not contain 0, indicating the existence of mediating effects in the impact of family socioeconomic status on elderly health. Moreover, the 95% confidence interval of the estimated mediation effect for the health-care status is [−0.014, 0.004] and contains 0, indicating the absence of the mediation effect.

## 5. Discussion

In the current study, based on the CLHLS in 2018, the total family income, the comprehensive years of education, and the comprehensive occupational rank before retirement of the elderly couples were synthesized into a family socioeconomic status index that was used as the explanatory variable using the entropy weight method, and the frailty index was used as a measurement of the comprehensive health status of the elderly. First, we established the multiple linear regression model and quantile regression models to analyze the effects of family socioeconomic status on the health status of the elderly and conducted a robustness test using the replaced explanatory variable. Then, the heterogeneity of the effect of family socioeconomic status on the health status of the elderly among different residential areas and at different ages was analyzed. Finally, the overall living status, leisure and recreation status, and health-care status of older adults were used as mediating variables to analyze their mediation effects in the influence of family socioeconomic status on elderly health.

Family socioeconomic status has a positive impact on the health status of the elderly. This result is same to those of other researchers [18,20,21,30]. Family socioeconomic status reflects the individual’s ability to obtain material and social resources [31]. Higher family socioeconomic status usually means higher total family income, education, and occupational rank [18]. Older adults with higher total family income tend to have better living conditions, participate in more leisure and entertainment activities to meet higher-lever needs, and purchase better health-care services to increase investment in health. Elderly families with higher years of education have acquired higher health awareness and literacy during their continuous learning and developed healthier living habits; they are more aware of various health risk factors and therefore more aware of their prevention, and are able to respond more quickly and effectively when they encounter diseases. Older households with higher occupational rank tend to have a higher proportion of pensions and have higher pensions; in addition, higher occupational rank tends to be accompanied by more available access to health-care services. Thus, higher family income, education, and occupational rank generally result in better health outcomes for older adults. Family socioeconomic status is a comprehensive reflection of the individual socioeconomic status of elderly couples, and it is the social class or status of elderly couples based on the family cooperation model. Therefore, the increase of family socioeconomic status can decrease the frailty index and promote the elderly health.

Due to the typical dual economic structure of urban and rural areas in China, the family socioeconomic status of the urban elderly is relatively higher (the average family socioeconomic status indices of the urban and rural elderly in the present study are, respectively, 0.237 and 0.126). Additionally, public health and medical resources in urban areas are more abundant and the allocation is more reasonable, while these conditions in rural areas are relatively poor. Under the influence of the law of diminishing marginal utility of the health production function [4,18], the family socioeconomic status of the urban elderly has lower influence on the frailty index. Some scholars also hold the analogous view [13,18]. In other words, when the family socioeconomic status changes by an equal amount, it has a higher impact on the health of the rural elderly.

Family socioeconomic status has a significantly positive influence on the health status of middle and lower age elderly, but not on higher age elderly, which is similar to the conclusion of other related studies [32,33]. When older adults reach the higher age, their physical functions continue to decline, and their health status becomes increasingly dependent on the individual and less influenced by other factors, including the family socioeconomic status, whereupon the effect of family socioeconomic status on the health of the higher age elderly is no longer significant.

A review study by Huang showed that there are four mediating pathways between socioeconomic status and health: material factors, lifestyle factors, psychosocial factors, and neighborhood [34]. Unlike that, we think that overall living status and leisure and recreation status have mediation effects in the influence of family socioeconomic status on the health status of the elderly, whereas health-care status has no mediation effect, which is different from the conclusion of other studies as well [12,13,14,35,36]. When older adults have a higher family socioeconomic status, on the one hand, their sources of living are often more abundant and their quality of life is usually higher, and thus their overall living status is better. They will pay more attention to direct investment in health. On the other hand, their health awareness tends to be higher, and it is more likely to increase the physical resistance through exercise and to relax by participating in various leisure and recreational activities. Therefore, overall living status and leisure and recreation status have mediation effects in the influence of family socioeconomic status on elderly health. With the expansion of medical insurance coverage and regular physical examination in China, not subject to the family socioeconomic status, more and more elderly people are able to be hospitalized in time when they fall ill and participate in annual routine medical checkups. As the basic public health services become more equalized, health-care status has no mediation effect in the effect of family socioeconomic status on elderly health.

There were several limitations to this research. First, the study only used the 2018 cross-sectional data, so we did not reveal the dynamic impact of family socioeconomic status on elderly health. Second, limited by the variables in CLHLS data, only 32 indicators were used to construct the frailty index. If more indicators can be obtained, the measurement of frailty index will be more accurate. Third, total family income and primary occupation rank before retirement of the elderly might be related to their health status, so there might be a reverse causality between family socioeconomic status and frailty index to some extent.

## 6. Conclusions

The main findings of this study are as follows:

1. This study explores the relationship between family socioeconomic status and the health status of the elderly in China from a comprehensive perspective. The improvement of the family socioeconomic status of the elderly will lower their frailty index, thereby promoting the improvement of their health.
2. The influence of family socioeconomic status on elderly health shows obvious urban–rural differences. Compared with the urban elderly, the family socioeconomic status of the rural elderly has a higher impact on the health of the elderly. As the public health and medical resources in urban areas are more abundant and the allocation is more reasonable, while these conditions in rural areas are relatively poor, the effect of promoting the health of the rural elderly by improving their family socioeconomic status is more significant.
3. The impacts of family socioeconomic status on the health of the elderly in different age groups are different. Family socioeconomic status has a significantly positive influence on the health status of middle and lower age elderly, but not on higher age elderly. As the elderly age, their physical functions continue to decline and their psychological status becomes more stable, and their health status becomes increasingly dependent on the individual and less influenced by other factors, including the family socioeconomic status.
4. Overall living status and leisure and recreation status have mediation effects in the influence of family socioeconomic status on the health status of the elderly, whereas health-care status has no mediation effect. Family socioeconomic status is to some extent the decisive factor of overall living status and leisure and recreation status. With the continuous equalization of China’s medical and health services, the mediating role of health-care status in the impact has become weaker.

Based on the above research, we propose the following countermeasures:

1. Improve the old-age insurance system. Expand the coverage of old-age insurance and improve pension benefits. At the same time, increase the transfer payment to the elderly and strengthen the economic security ability for them to improve their living conditions.
2. Promote healthy aging of the elderly. Enlarge the enrollment scale of universities for the elderly and enrich the teaching contents. In particular, health education for the elderly should be strengthened to improve their health awareness and health literacy.
3. Accelerate the implementation of the delayed retirement policy and gradually postpone the retirement age of the elderly. At the same time, accelerate the development of human resources of the elderly to delay the decline of their occupational rank.
4. Improve the medical insurance system. Expand the coverage of medical insurance and increase the reimbursement ratio of medical expenses. At the same time, improve the level of health-care technology and improve the health-care status of the elderly.
5. Promote active aging of the elderly. On the one hand, the government and society should provide more leisure and recreational activities, places, and facilities; on the other hand, the elderly should be encouraged to participate in more recreational activities and other social activities.
6. Pay more attention to the key elderly populations. For example, public health policies should be strengthened for the rural elderly and the higher age elderly.

## Figures and Tables

**Table 1:** Variables and data statistics.

Continuous Variables	Mean	Standard Deviation	Min	Max
Health status	0.211	0.144	0	1
Family socioeconomic status	0.189	0.197	0	1
Age	83.872	11.488	60	117
Number of surviving children	3.437	1.786	0	11
**Categorial Variables**	**Categories**	***n***	**Percentages (%)**	**Average Frailty Index**
Gender	Female	4228	55.6	0.236
	Male	3371	44.4	0.181
Marital status	Without spouse	4519	59.5	0.252
	With spouse	3080	40.5	0.152
Residential area	Rural	3266	43.0	0.205
	Urban	4333	57.0	0.216
Living with family members	No	1623	21.4	0.214
	Yes	5976	78.6	0.210
Living in an institution	No	7326	96.4	0.207
	Yes	273	3.6	0.320
Old-age insurance	Do not have	3807	50.1	0.219
	Have	3792	49.9	0.203
Medical insurance	Do not have	1049	13.8	0.235
	Have	6550	86.2	0.207
Overall living status	Bad	2645	34.8	0.250
	Good	4954	65.2	0.191
Leisure and recreation status	Bad	7011	92.3	0.220
	Good	588	7.7	0.112
Health-care status	Bad	2438	32.1	0.261
	Good	5161	67.9	0.188

**Table 2:** Regression results of the impact of family socioeconomic status on elderly health.

Variables	Model 1	Model 2	Model 3	Model 4	Model 5	Model 6
	OLS	Q10	Q25	Q50	Q75	Q90
Explanatory variable						
Family socioeconomic status	−0.05 ***	−0.041 ***	−0.058 ***	−0.058 ***	−0.055 ***	−0.067 ***
	(0.009)	(0.008)	(0.007)	(0.009)	(0.012)	(0.017)
Control variables						
Gender	−0.030 ***	−0.017 ***	−0.023 ***	−0.028 ***	−0.032 ***	−0.039 ***
(Female)	(0.003)	(0.003)	(0.003)	(0.003)	(0.004)	(0.005)
Age	0.006 ***	0.003 ***	0.004 ***	0.00 6***	0.007***	0.008 ***
	(0.000)	(0.000)	(0.000)	(0.000)	(0.000)	(0.000)
Marital status	−0.012 ***	0.001	−0.004	−0.015 ***	−0.021 ***	−0.022 ***
(Without spouse)	(0.004)	(0.003)	(0.004)	(0.004)	(0.006)	(0.008)
Residential area	0.007 **	0.002	0.006 *	0.004	0.007	0.013 **
(Rural)	(0.003)	(0.003)	(0.003)	(0.003)	(0.004)	(0.006)
Living with family members	0.031 ***	0.012 ***	0.018 **	0.031 ***	0.037 ***	0.040 ***
(No)	(0.004)	(0.003)	(0.004)	(0.005)	(0.006)	(0.009)
Living in an institution	0.106 ***	0.044 ***	0.058 ***	0.100 ***	0.145 ***	0.173 ***
(No)	(0.010)	(0.011)	(0.010)	(0.019)	(0.018)	(0.027)
Number of surviving children	−0.002 **	−0.002 **	−0.002 **	−0.003 ***	−0.002	0.001
	(0.001)	(0.001)	(0.001)	(0.001)	(0.001)	(0.002)
Old-age insurance	−0.001	−0.002	0.000	−0.003	−0.005	0.004
(Do not have)	(0.003)	(0.003)	(0.003)	(0.003)	(0.005)	(0.006)
Medical insurance	−0.012 ***	−0.002	−0.006	−0.014 **	−0.013 **	−0.013
(Do not have)	(0.004)	(0.003)	(0.004)	(0.006)	(0.006)	(0.010)
Constant	−0.260 ***	−0.148 ***	−0.192 ***	−0.264 ***	−0.317 ***	−0.290 ***
	(0.014)	(0.017)	(0.013)	(0.014)	(0.022)	(0.027)
R^2^/Pseudo R^2^	0.310	0.081	0.126	0.196	0.234	0.207

The robust standard errors are in parentheses in Model 1, and the statistic for measuring goodness-of-fit is R^2^. The bootstrap standard errors are in parentheses in Models 2–6, with a sample size of 100, and the statistic for measuring goodness-of-fit is Pseudo R^2^. * *p* < 0.1, ** *p* < 0.05, *** *p* < 0.01.

**Table 3:** Regression results after replacing the explanatory variable.

Variables	Model 7	Model 8	Model 9	Model 10	Model 11	Model 12
	OLS	Q10	Q25	Q50	Q75	Q90
Replaced family socioeconomic status	−0.070 ***	−0.050 ***	−0.071 ***	−0.075 ***	−0.081 ***	−0.100 ***
	(0.011)	(0.009)	(0.008)	(0.011)	(0.015)	(0.024)
Constant	−0.258 ***	−0.147 ***	−0.192 ***	−0.261 ***	−0.310 ***	−0.284 ***
	(0.014)	(0.015)	(0.014)	(0.016)	(0.021)	(0.024)
R^2^/Pseudo R^2^	0.311	0.082	0.127	0.197	0.235	0.209

The robust standard errors are in parentheses in Model 7, and the statistic for measuring goodness-of-fit is R^2^. The bootstrap standard errors are in parentheses in Models 8–12, with a sample size of 100, and the statistic for measuring goodness-of-fit is Pseudo R^2^. *** *p* < 0.01. The control variables in each model have been controlled.

**Table 4:** Results of multiple linear regression (with explanatory variable) by residential area.

Variables	Model 13		Model 14	
	Urban		Rural	
	Coefficient	Standard Deviation	Coefficient	Standard Deviation
Family socioeconomic status	−0.043 ***	0.011	−0.088 ***	0.018
Constant	−0.282 ***	0.019	−0.215 ***	0.021
*n*	4333		3266	
F value	228.130 ***		163.550 ***	
R^2^	0.313		0.309	

*** *p* < 0.01. The control variables in each model have been controlled.

**Table 5:** Results of multiple linear regression (with explanatory variable) by age.

Variables	Model 15		Model 16		Model 17	
	60–69 Years Old		70–79 Years Old		80 Years Old and Above	
	Coefficient	Standard Deviation	Coefficient	Standard Deviation	Coefficient	Standard Deviation
Family socioeconomic status	−0.086 ***	0.018	−0.094 ***	0.014	−0.009	0.014
Constant	0.046	0.115	−0.124 **	0.058	−0.423 ***	0.028
*n*	976		1977		4646	
F Value	10.910 ***		12.880 ***		144.100 ***	
R^2^	0.116		0.080		0.220	

** *p* < 0.05, *** *p* < 0.01. The control variables in each model have been controlled.

**Table 6:** Results of multiple linear regression adding mediating variables.

Variables	Model 18		Model 19		Model 20	
	Coefficient	Standard Deviation	Coefficient	Standard Deviation	Coefficient	Standard Deviation
Explanatory variable						
Family socioeconomic status	−0.030 ***	0.009	−0.035 ***	0.009	−0.049 ***	0.009
Mediating variables						
Overall living status	−0.071 ***	0.003				
Leisure and recreation status			−0.054 ***	0.004		
Health-care status					−0.032 ***	0.003
Constant	−0.243 ***	0.013	−0.243 ***	0.014	−0.217 ***	0.014
F value	392.380 ***		348.410 ***		332.720 ***	
R^2^	0.363		0.319		0.319	

*** *p* < 0.01. The control variables in each model have been controlled.

**Table 7:** Estimated results of mediating effects.

Mediating Variables	Mediating Effect	Standard Deviation	95% Confidence Interval	Yes/No
Overall living status	−0.095	0.013	[−0.116, −0.074]	Yes
Leisure and recreation status	−0.155	0.017	[−0.184, −0.127]	Yes
Health-care status	−0.005	0.006	[−0.014, 0.004]	No

We set rho as 0, alpha as 0.1, and type as “mc” in the R software.
